# Occurrence of adult‐onset Still's disease after coronavirus disease 2019 BNT162B2 vaccination in a patient with ulcerative colitis: A case report and review of literature

**DOI:** 10.1002/ccr3.8298

**Published:** 2023-12-05

**Authors:** Takahiro Kobayashi, Kenichi Hashimoto, Yasuyoshi Kusanagi, Yuji Tanaka

**Affiliations:** ^1^ Department of General Medicine National Defense Medical College Tokorozawa Japan; ^2^ Department of Internal Medicine, Division of Hematology and Rheumatology National Defense Medical College Tokorozawa Japan

**Keywords:** adult‐onset Still's disease, COVID‐19 vaccine, SARS‐CoV2, ulcerative colitis

## Abstract

**Key Clinical Message:**

We report an extremely rare occurrence of adult‐onset Still's disease (AOSD) in a patient with ulcerative colitis. The possibility of autoinflammatory conditions such as AOSD should be considered when evaluating or treating symptoms suspected as side effects of the severe acute respiratory syndrome coronavirus 2 vaccination, regardless of the associated comorbidities.

**Summary:**

A woman in her 50s with a history of stable ulcerative colitis (UC) for 20 years, managed using salazosulfapyridine, presented with migratory rashes, spiking fever, edema, and joint pain that started 1 week after receiving the BNT162B2 mRNA vaccine against severe acute respiratory syndrome coronavirus 2 (SARS‐CoV‐2). Laboratory tests revealed extremely high serum ferritin levels. The patient was diagnosed with adult‐onset Still's disease (AOSD) based on the relevant classification criteria after ruling out other diseases. Detection of high levels of interleukin‐18, an inflammatory cytokine related to AOSD, supported the diagnosis. Nonsteroidal anti‐inflammatory drug monotherapy alone resulted in significant improvements in both the abovementioned symptoms and the elevated inflammatory marker levels. AOSD in a patient with UC is extremely rare. Only one case of AOSD with UC was reported before the coronavirus disease 2019 era. This case indicates that SARS‐CoV‐2 vaccination can trigger a hyperinflammatory response, classified as AOSD, in a patient with UC, which is extremely rare.

## Graphical abstract

Salmon‐pink maculopapular rashes with abrasions were observed on lower legs, on her first visit of the hospital after SARS‐CoV‐2 vaccination.

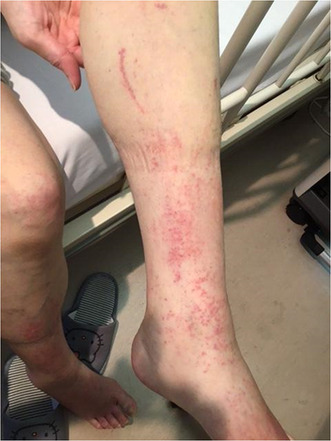

## 1 INTRODUCTION

Adult‐onset Still's disease (AOSD) is a systemic autoinflammatory illness characterized by recurrent spiking fever, fleeting salmon‐pink skin rashes, and polyarthritis.^1^ Although the etiology of AOSD remains elusive, dysregulation of immune cells, particularly macrophages and T cells, and the release of various proinflammatory cytokines such as interleukin (IL)‐18 may play significant roles in its development.^2^ Reportedly, vaccination against severe acute respiratory syndrome coronavirus 2 (SARS‐CoV‐2) could induce a hyperinflammatory state by triggering the production of spike proteins^3^; however, the correlation between SARS‐CoV‐2 vaccination and hyperinflammation remains to be elucidated. Moreover, the coexistence of AOSD with ulcerative colitis (UC), an inflammatory intestinal disease involving the colon, is extremely rare. Only one case of AOSD with UC has been reported to date.^4^ Herein, we report a case of a patient with stable UC who developed AOSD after receiving the first dose of a SARS‐CoV‐2 vaccine and was treated using nonsteroidal anti‐inflammatory drugs (NSAIDs) alone.

## 2 CASE HISTORY/EXAMINATION

A woman in her 50s with a history of stable UC for over 20 years presented to our hospital with a complaint of migratory joint pain. One week before presentation, she experienced arthralgia in her elbow and right hip, accompanied by throat pain, skin rashes, and an intermittent daily fever that started abruptly. She developed intermittent migratory rashes on her limbs following daily fever spikes (up to approximately 38°C), demonstrating a possible temporal relevance between the rashes and fever. Bilateral ankle pain and loss of appetite followed, leading her to seek medical care at a hospital, where acetaminophen (400 mg as needed) was prescribed. Although the joint pain in her elbows and hip disappeared, acetaminophen was not effective in relieving the ankle pain. Furthermore, she experienced bilateral wrist pain and visited another hospital for further pain relief. Celecoxib (100 mg two times/day) was prescribed and was partially effective for pain relief. She was subsequently referred to our hospital for further treatment.

The patient had no significant medical history other than UC, which was diagnosed in her 30s and managed using salazosulfapyridine (3000 mg per day). The patient showed no acute deterioration under this treatment, and her annual colonoscopy showed no flares in her intestines. Her family history was insignificant, and she had no allergies, travel history, history of insect exposure, or history of sexual activity that may explain the migratory rashes and arthralgia.

**FIGURE 1:**
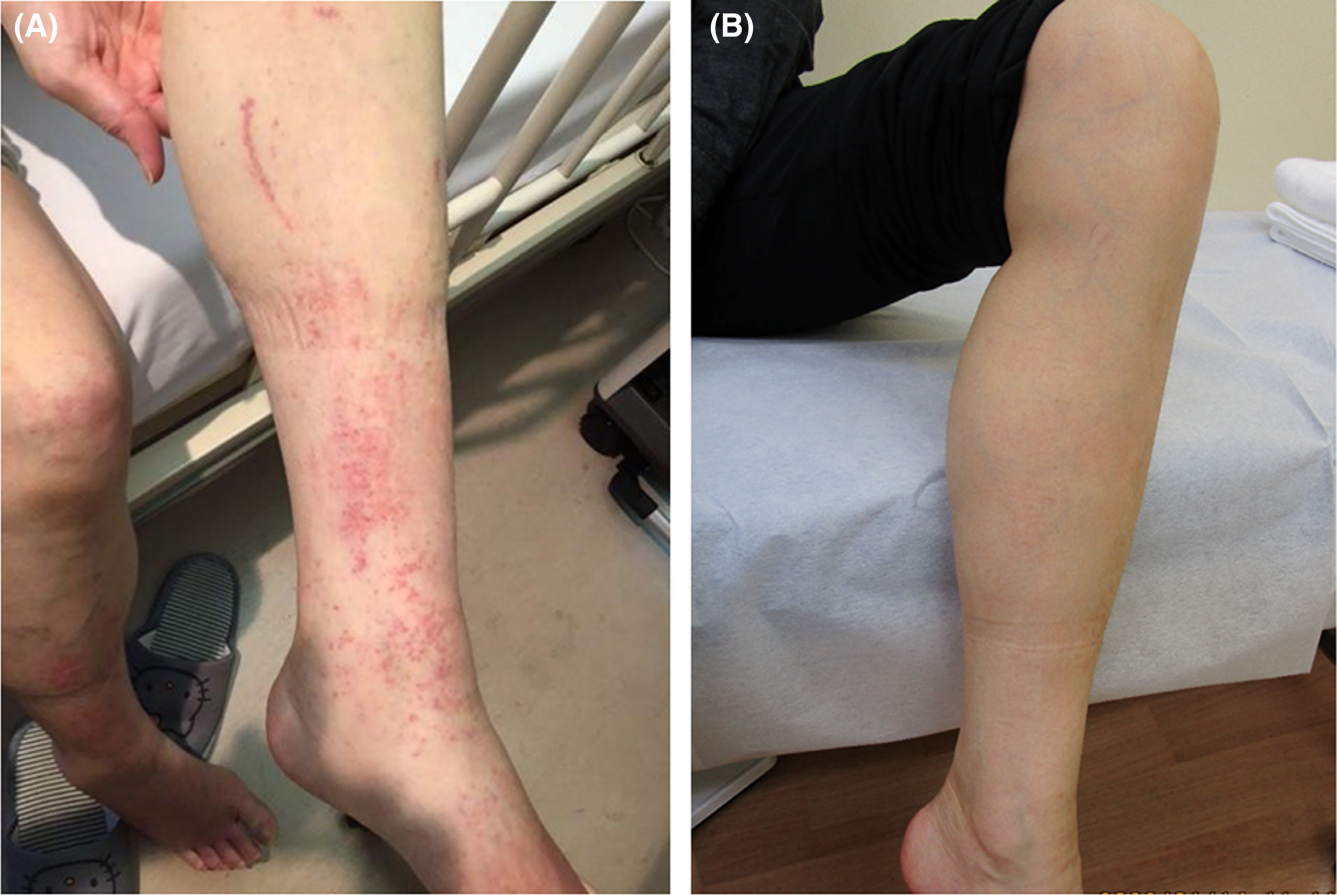
(A) Salmon‐pink rashes with scratch dermatitis on the lower left leg. (B) Skin at 3 months following treatment.

On her first visit, she presented with an afebrile status accompanied by tachycardia (105 beats per minute). Salmon‐pink maculopapular rashes with abrasions were observed on her forearms, abdomen, and lower legs (Figure 1A). Her anterior neck lymph nodes showed painless lymphadenopathy (maximum diameter, 15 mm). She reported tenderness in multiple joints, including the wrists and ankles, and the pain intensified with active joint movement.

## 3 DIFFERENTIAL DIAGNOSIS, INVESTIGATIONS, AND TREATMENT

Bacterial infection was one of the major differential diagnoses. Laboratory tests performed on admission revealed leukocytosis (16,700 cells/mm^3^) with neutrophilia (14,863 cells/mm^3^), a C‐reactive protein level of 35.2 mg/dL, an erythrocyte sedimentation rate of 98 mm/h, and a ferritin level of 1651.5 ng/mL.

Ceftriaxone treatment, as an empiric antibiotic therapy, was started immediately after admission because the nephrologists considered the risk of infection‐related acute glomerular nephritis owing to the presence of bacteria on urinalysis. However, we discontinued antibiotic therapy 5 days later after confirmation of negative blood culture results and rapid improvement in the urine sediment with no pathognomonic casts, which are not compatible with the indications of acute kidney injury that requires a kidney biopsy. The result of the urinalysis showed *Lactobacillus* species, interpreted as normal bacterial flora. An anti‐streptolysin titer showed negative results. The *Treponema pallidum* hemagglutination test, the rapid plasma reagin test, and interferon gamma assays for tuberculosis showed negative results. The patient did not have any exposure to insects and had not visited Lyme disease‐endemic areas.

In addition, viral causes were also included in the differential diagnosis. The SARS‐CoV2 rapid antigen test results were negative. The human immunodeficiency virus test result was negative; further, the patient had not been sexually active for years and had never used intravenous drugs. Hepatitis B surface antigen and anti‐human parvovirus B19 immunoglobin M were both negative.

Malignant causes were listed under the “must rule out” category. Positron emission tomography‐computerized tomography (PET‐CT) indicated 18F‐fluorodeoxyglucose uptake in the lymph nodes (neck, axillary, mediastinal, inguinal, and liver), spleen, and bone marrow. Axillary lymph node biopsy revealed reactive lymphoid follicular hyperplasia with no malignant changes. The colonoscopy, mammography, and Pap smear test results were all within normal limits.

Autoimmune‐related assessments, including antinuclear antibody, rheumatoid factor, anti‐citrullinated peptide antibody, anti‐double‐stranded DNA antibody, anti‐SS‐A antibody, and anti‐neutrophil cytoplasmic antibody tests, showed normal results and did not meet the classification criteria for any autoimmune‐related diseases.

Spondyloarthritis (SpA) associated with UC was one of the differential diagnoses. However, the patient's joint pain was not classified as arthritis, enthesitis, or dactylitis, thus not meeting the classification criteria of peripheral spondyloarthritis.^5^ The HLA‐B‐27 test result was also negative. Regarding the possibility of axial SpA, the absence of back pain and the patient's age being >45 years did not meet the classification criteria.

Diagnosis of AOSD requires a thorough clinical investigation to rule out other possible inflammatory diseases, including malignancies, infections, and other rheumatic diseases. Malignant lymphoma is a fatal condition that comes first as a differential diagnosis, mimicking AOSD. However, biopsies of the patient's lymph nodes and the absence of malignant skin cells showed no signs of malignant lymphoma. Results of a PET‐CT scan and cancer screening tests (colonoscopy, mammography, and Pap smear tests) did not suggest any solid malignant tumor. Finally, 12 days after admission, the patient's ferritin levels increased to 2382.6 ng/mL, and her serum IL‐18 levels (131,000 pg/mL) were extremely high.

The patient's medical history, physical examination findings, and lab test results indicated that the diagnostic criteria for AOSD were met (Yamaguchi's criteria^6^; three of four major criteria [arthralgia/arthritis, typical rash, and leukocytosis] and four of five minor criteria [sore throat, lymphadenopathy, abnormal liver function test results, and negative rheumatoid factor and antinuclear antibody assays] were met). Furthermore, her serum IL‐18 levels increased to 131,000 pg/mL, and her ferritin levels increased as her symptoms worsened, which strongly supported the diagnosis of AOSD. PET‐CT findings showing high absorption in the bone marrow demonstrated a typical pattern for AOSD. Thus, new‐onset Still's disease was considered the most likely diagnosis.

We initially considered restarting acetaminophen instead of NSAIDs to relieve the patient's joint pain, even though NSAIDs are generally contraindicated for patients with UC because they may trigger a relapse.^7^, ^8^ We finally decided to continue the NSAID therapy, considering that the severe arthralgia that was affecting her motor function was improving with the use of celecoxib.

We started the patient on loxoprofen and gradually increased its dose while continuing salazosulfapyridine and confirming the absence of hematochezia or abdominal pain, a sign of relapsing UC. If treatment failed, we planned to intensify the dose of steroid or tocilizumab treatment, which is reportedly effective for AOSD. The patient responded to the NSAID therapy with loxoprofen (180 mg daily [maximum dose]) and showed a gradual and significant improvement in the fever, rashes, and fatigue. She was subsequently discharged from our hospital within 3 weeks.

## 4 OUTCOME AND FOLLOW‐UP

**FIGURE 2:**
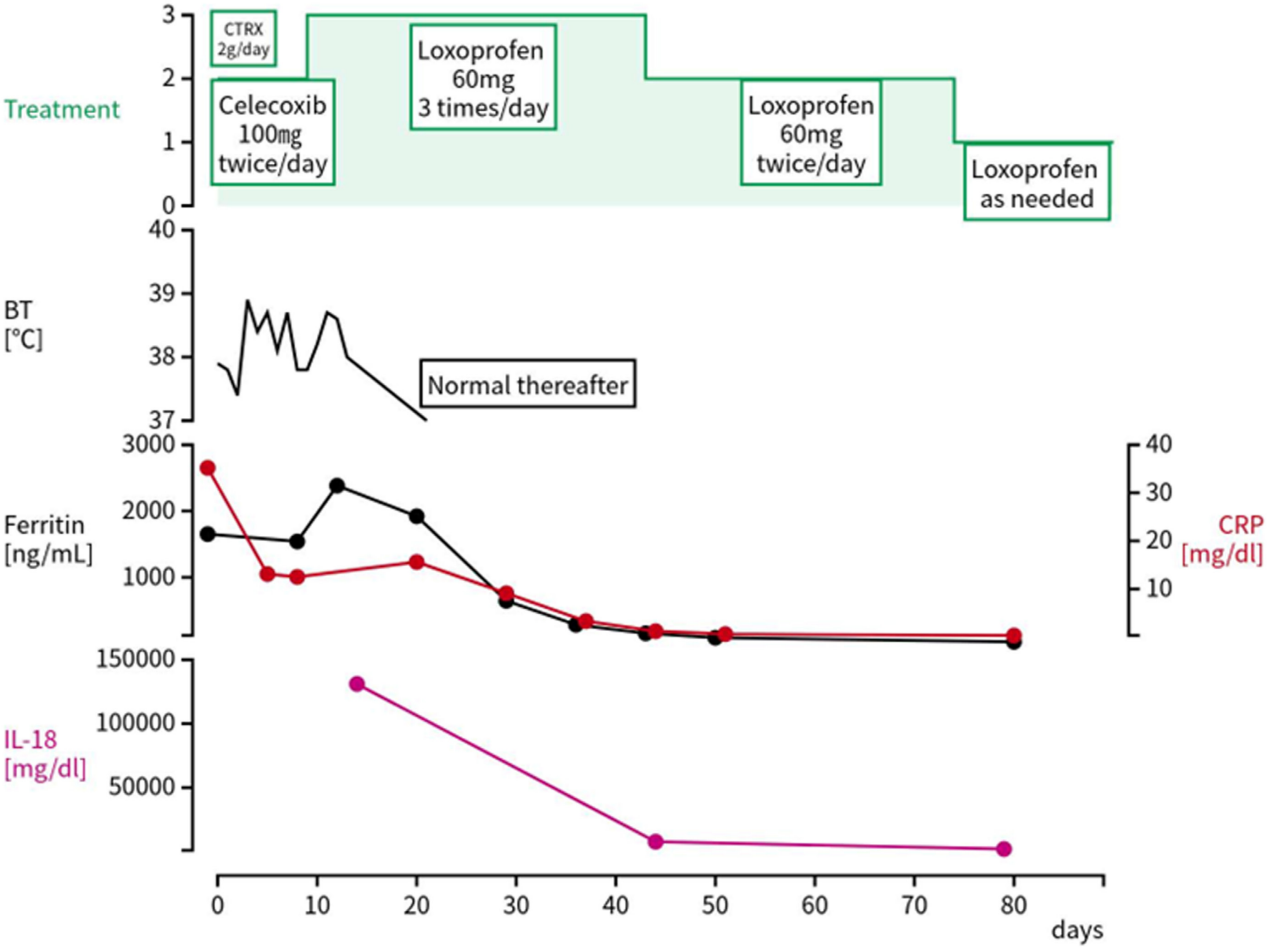
Clinical course of the case, including data on the patient's body temperature, laboratory data, and treatment data. BT, body temperature; CTRX, ceftriaxone; CRP, C‐reactive protein; IL, interleukin.

Figure 2 shows the clinical course of the case. NSAID therapy was significantly and continuously effective in relieving the patient's joint symptoms. She was discharged 9 days after admission following significant improvement in her symptoms. We continued the usual recommended dose of NSAIDs (loxoprofen 60 mg three times/day), and no side effects or reactivation of UC, bloody diarrhea, or abdominal pain occurred. Follow‐up endoscopy performed 9 months later showed no abnormalities in the patient's large intestine.

Considering the significant improvements in arthralgia, edema, and fever, as well as the normalized inflammatory indicators within 3 months of NSAID monotherapy (loxoprofen 60 mg three times/day), administration of steroids or tocilizumab was deemed unnecessary. The rashes also disappeared within 3 months (Figure 1B). We gradually decreased the dose of loxoprofen, considering the possible risk of recurrent UC, and finally discontinued the NSAID therapy within 12 months. All joint symptoms, fever, and rashes disappeared, and the patient's ferritin, C‐reactive protein, and IL‐18 levels normalized at the completion of follow‐up in 1 year.

Regarding the anti‐SARS‐CoV2 vaccination status, the patient commented as follows (the following perspective was translated by the author as English is not the patient's native language): “I was worried about the joint symptoms, as they interfered with my daily activities. However, pain killers relieved my joint pain gradually but significantly. The possible relationship between my disease and coronavirus disease 2019 vaccination is a bit worrisome, and I contemplate whether I should receive another shot. I will consider the consensus of the doctors and the prevalence of coronavirus disease 2019 when deciding”.

## 5 DISCUSSION

This article reports the development of AOSD following mRNA SARS‐CoV‐2 vaccination in a patient with UC. This phenomenon suggests that the vaccine can cause a hyperinflammatory response. Multiple cases of new‐onset AOSD following the SARS‐CoV‐2 vaccination have been documented.^9^, ^10^, ^11^, ^12^, ^13^, ^14^ Flares of AOSD have also been reported, suggesting a possible correlation between the disease and SARS‐CoV‐2 vaccines.^15^, ^16^, ^17^, ^18^ The reported cases of a new occurrence of AOSD after SARS‐CoV‐2 vaccination, including the present case, are summarized in Table 1.

**TABLE 1:** A review of case reports and case series: New‐onset adult‐onset Still's disease following the patient's first SARS‐CoV‐2 vaccination.

Age (years) and sex (M/F)	SARS‐CoV‐2 vaccine (type, name)	Past medical history	Onset (days/weeks/months after vaccination until any symptoms started)	Serum ferritin (ng/mL)	Serum IL‐18 (pg/mL)	Medication	Published years	Reference
22 M	mRNA, BNT162B2	None	13 days	54,921	NA	Methylprednisolone, Dexamethasone, Anakinra, and IVIG	2022	^3^
36 M	Vector, ChAdOx1 nCoV‐19	None	1 day	1482	NA	Methylprednisolone	2021	^9^
36 F	mRNA, BNT162B2	None	10 days	4312	NA	Methylprednisolone and Tocilizumab	2021	^10^
53 M	Vector, ChAdOx1 nCoV‐19	None	10 weeks	3140	NA	Prednisone	2021	^11^
20 F	Vector, ChAdOx1 nCoV‐19	None	10 days	11,491	NA	Naproxen	2022	^12^
47 F	Vector, ChAdOx1 nCoV‐19	None	3 weeks	404	NA	Methotrexate and Tocilizumab	2022	^12^
35 F	Vector, ChAdOx1 nCoV‐19	None	3 months	>100,000	NA	Methylprednisolone	2022	^12^
59 F	mRNA, BNT162B2	NA	10 days	11,491, IL‐18	≥5000	Methylprednisolone, Prednisolone, Tocilizumab, and Cyclosporin	2023	^13^
35 M	mRNA‐1273	NA	24 days	1263	NA	Prednisolone	2023	^13^
65 M	Vector, ChAdOx1 nCoV‐19	Psoriatic arthritis	1 day	1550	NA	Prednisone and Anakinra	2022	^14^
50 F	mRNA, BNT162B2	UC	21 days	2383	131,000	Loxoprofen	2022	Our case

Abbreviations: F, female; IL, interleukin; IVIG, intravenous immunoglobulin; M, male; NA, not available; NS, not significant; SARS‐CoV‐2, severe acute respiratory syndrome coronavirus 2; UC, ulcerative colitis.

We performed a literature review to identify case reports about new‐onset (not flare) AOSD following the SARS‐CoV‐2 vaccination. This review was based on the PubMed, Embase, Medline, and Journals@Ovid databases. Full text was acquired by entering the terms “adult‐onset Still's disease” and “SARS‐CoV2 vaccination.” The review included articles in English, and case reports and case series were identified. Seven articles with 10 cases in total, all of which were published during 2021–2023, were finally collected. All of them were diagnosed using the Yamaguchi criteria and included details on the onset of the disease after the SARS‐CoV2 vaccination, type of the vaccine, and patients' ferritin levels.

The ages of the patients in these cases ranged from 22 to 65 years, and they had few comorbidities. Both vaccine types, including mRNA and vector vaccines, provoked new‐onset AOSD. These patients presented with fever, skin rashes, and musculoskeletal symptoms. All patients had new‐onset AOSD, which was treated using NSAIDs, steroids, tocilizumab, anakinra, and intravenous immunoglobulin. Eight cases required immunosuppressive therapies with steroids or tocilizumab. Two cases, including ours, were interpreted as being those of mild AOSD and were treated with NSAIDs. All patients recovered after treatment. Some hyperinflammatory responses after vaccination can be self‐limiting; however, cases similar to those listed in Table 1, wherein the symptoms meet the classification criteria for AOSD and improve after treatment, should be carefully assessed without dismissing the symptoms as simple side effects of the SARS‐CoV‐2 vaccine. Skin rashes, fever, and musculoskeletal symptoms are usually not specific to a certain diagnosis and can be interpreted as side effects of the vaccine shot. However, all the patients mentioned above presented with worsening symptoms for weeks after the SARS‐CoV2 vaccination. Considering the self‐limiting characteristics of vaccine side reactions, worsening and long‐lasting symptoms should be investigated, highlighting the Yamaguchi criteria.^6^ On average, the symptoms of AOSD in the reported cases generally started 3 weeks after vaccination. In addition, patients should be appropriately notified when the diagnosis is made. To the best of our knowledge, the present case is the first reported case of a patient with UC who developed AOSD after SARS‐CoV‐2 vaccination and was treated using only NSAIDs. The distinguishing characteristics of the present case are the presence of UC as a comorbidity and the positive response to NSAID treatment, even with extremely high levels of IL‐18, which is suggested to be an inflammatory marker of AOSD^2^ and is correlated with the severity of active AOSD.^19^ Patients with AOSD show extremely high IL‐18 levels compared with those of patients with other inflammatory diseases.^19^ Sequential measurement of IL‐18 levels may be useful in guiding treatment, as they can reflect disease activity in AOSD.^20^ A clinical practice guideline suggests that the IL‐18 level in addition to ferritin and C‐reactive protein levels can be used to evaluate the extent of inflammation in Still's disease.^21^ The guideline also indicates that NSAIDs can be used in addition to the mainstay treatment comprising systemic steroids, which can be used to alleviate symptoms with mild AOSD.^21^

In the present case, the positive response to NSAID treatment in the presence of high IL‐18 levels after SARS‐CoV‐2 vaccination supports the diagnosis of AOSD. The involvement of inflammatory cytokines, which can be induced by postvaccination new‐onset AOSD or AOSD flares, may imply that the common background etiology includes inflammasomes and hyperinflammation through macrophage activation and proinflammatory cytokines, such as IL‐6, IL‐18, and tumor necrosis factor.

Adult‐onset Still's disease is a rare comorbidity in patients with UC. Only one case of Crohn's disease (CD) with AOSD has been reported.^4^ Multiple factors that are likely relevant to the coexistence of AOSD and inflammatory bowel disease include intestinal microbiota, mucosal barrier, genetic susceptibility, and environmental factors; however, no definitive etiology has yet been clarified.^22^ Although the detailed mechanism remains unknown, several studies supporting an association between the arthritis‐related AOSD and inflammatory bowel disease (IBD) have been reported.^4^ Moreover, it has been reported that 81% of patients with juvenile idiopathic arthritis, a disease related to AOSD, had CD lesions, as revealed by ileocolonoscopy.^23^ In addition, a report from a Finnish group showed a significantly higher incidence of arthritis in pediatric‐onset IBD patients (5.4% vs. 0.2%) than in controls.^24^ It should be noted here that these reports did not represent AOSD per se (with UC). However, from a molecular biological point of view, both AOSD and IBD share the involvement of tumor necrosis factor‐α as a cytokine.^25^ Nevertheless, the present case indicates that even though the coexistence of UC and AOSD is rare, SARS‐CoV‐2 vaccines may trigger a specific inflammatory process in patients with UC. As mentioned in the differential diagnosis, UC flares are one of the important differential diagnoses. In fact, according to a Japanese cohort study tracking 188 patients with UC and 119 with CD, eight patients with UC and one with CD experienced disease flares, defined as a partial Mayo score of ≥3.^26^ A flare‐up of UC bowel symptoms might be related to SARS‐CoV2 vaccination. However, in that study, musculoskeletal symptoms were not analyzed. To the best of our knowledge, the relationship between SARS‐CoV‐2 vaccination and musculoskeletal symptoms in UC has not been reported. Furthermore, this patient did not have a flare‐up of bowel UC symptoms, as confirmed by lower endoscopy. A flare‐up of extra‐bowel UC symptoms with a stable bowel condition can be regarded as extremely rare. On the other hand, SpA with IBD can occur independently of the activity of IBD. However, this patient's condition did not meet the classification criteria for SpA, as described in the differential diagnosis, making the diagnosis of UC with SpA also unlikely. The extremely high IL‐18 level and specific finding of AOSD^19^ also do not support the classification of SpA. Moreover, as observed in another case report with a past medical history of psoriatic arthritis, one of the SpA subtypes, the case was diagnosed as AOSD and not as SpA.^14^

The phenomenon observed in our patient suggests that her hyperinflammatory status suppressed by salazosulfapyridine may have been substituted by an autoinflammatory condition triggered by SARS‐CoV‐2 vaccination, not classified as SpA.

In conclusion, this is the first report of the occurrence of AOSD in a patient with UC. The possibility of autoinflammatory conditions such as AOSD should be considered when evaluating or treating symptoms suspected to be side effects of SARS‐CoV‐2 vaccination, regardless of the comorbidities associated with such symptoms.

## AUTHOR CONTRIBUTIONS

**Takahiro Kobayashi:** Conceptualization; data curation; project administration; writing – original draft. **Kenichi Hashimoto:** Data curation; project administration; supervision; writing – review and editing. **Yasuyoshi Kusanagi:** Supervision; writing – review and editing. **Yuji Tanaka:** Supervision; writing – review and editing.

## FUNDING INFORMATION

This work was supported by the Fukuda Foundation for Medical Technology (2019).

## CONFLICT OF INTEREST STATEMENT

The authors report no conflict of interest.

## ETHICS STATEMENT

The study was approved by the Competent Authorities and Ethics Committees of National Defense Medical College (approval no. 4609). Written informed consent was obtained from all patients.

## CONSENT

Written informed consent was obtained from the patient to publish this report in accordance with the journal's patient consent policy.

## Data Availability

Not applicable.
